# Palatal Pyogenic Granuloma Suspected of Malignant Tumor: A Case Report and Literature Review

**DOI:** 10.7759/cureus.74284

**Published:** 2024-11-23

**Authors:** Tougo Tanabe, Taku Kimura, Ken-ichiro Sakata, Aya Yanagawa-Matsuda, Yoshimasa Kitagawa

**Affiliations:** 1 Department of Oral Diagnosis and Medicine, Hokkaido University, Sapporo, JPN; 2 Department of Vascular Biology and Molecular Pathology, Hokkaido University, Sapporo, JPN

**Keywords:** diabetes, malignancies, minor salivary gland tumor, palate, pyogenic granuloma

## Abstract

Pyogenic granuloma is a nonneoplastic inflammatory reactive hyperplasia commonly found in the soft tissues of the skin and oral mucosa. Oral pyogenic granulomas are usually found on the lips, gingiva, and tongue, but rarely on the palate. Surgical excision is the standard treatment for oral pyogenic granulomas. If it occurs on the palate, it is important to differentiate it from salivary gland tumors. Additionally, managing the disease with attention to recurrence is important. In this report, we present the case of a 66-year-old man who presented to the Department of Oral Diagnosis and Medicine at Hokkaido University Hospital with a mass lesion between the soft and hard palates. The patient was followed up for one year, but the lesion gradually enlarged and was accompanied by hemorrhage. Because of the atypical clinical course of the lesion, it was excised with a 7 mm safety margin, considering the possibility of palatal malignancy. Histopathological examination revealed oral pyogenic granuloma. When treating palatal tumors, clinicians should consider the possibility of minor salivary gland tumors, including malignancies.

## Introduction

Pyogenic granulomas are granulomatous and occur on the skin and mucosa [1]. Oral pyogenic granulomas usually develop on the lips, gingiva, and tongue [2] but rarely on the palate [3]. The typical clinical presentation of a pyogenic granuloma is a reddish, pedunculated, sessile, smooth-lobulated soft tissue mass. The standard treatment for oral pyogenic granulomas is surgical excision. However, their recurrence is occasionally observed [4]. Hence, excision from the base of the lesion is required [5]. Regarding the risk factors for developing oral pyogenic granulomas, mucosal trauma caused by ill-fitting dentures has been reported to be the main local risk factor [6,7]. However, systemic factors, such as endocrine imbalance, diabetes mellitus, and pregnancy, are risk factors for the development of this disease [8,9]. These systemic factors contribute to the decline of patients’ immune systems, making them susceptible to reactive proliferation in response to local factors. Hence, combining these systemic and local factors contributes to disease onset. When the size of the lesions gradually increases, malignant tumors should be considered [10]. In this case report, we present a case of oral pyogenic granuloma in which surgical resection was performed with an extensive safety margin because the possibility of malignancy could not be ruled out.

## Case presentation

**Figure 1:**
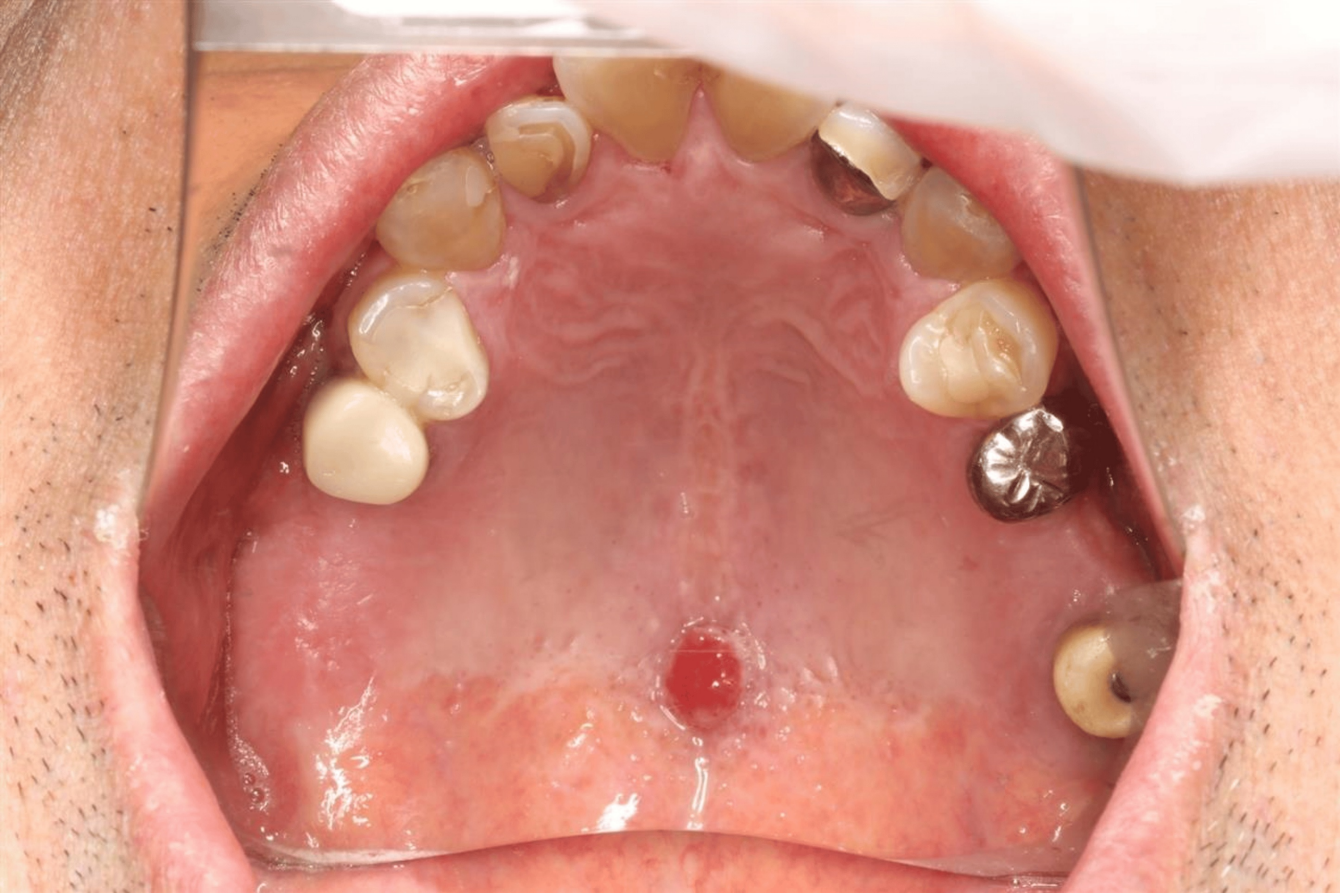
A 5 × 7 mm mass between the soft and hard palates

A 66-year-old man was pointed out a mass lesion between the soft and hard palates by a dentist at the nearest dental clinic where he received maintenance treatment. He was deemed to require further examination and treatment and was referred to the Department of Oral Diagnosis and Medicine at Hokkaido University Hospital. The patient had a medical history of diabetes mellitus (hemoglobin A1c: 6.5%). Two years before the first presentation to our department, he noticed the lesion and experienced recurrence several times. The extraoral examination revealed no apparent palpable cervical lymph nodes. The intraoral examination revealed a pedunculated mass of 5 × 7 mm in size between the soft and hard palates. The mass was well-demarcated, painless, red, and soft (Figure 1).

**Figure 2:**
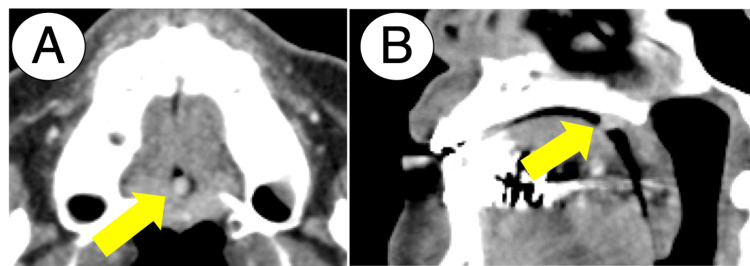
Contrast-enhanced computed tomography: (A) axial view and (B) coronal view

Contrast-enhanced computed tomography revealed the mass lesion with a high contrast signal. Additionally, contrast-enhanced computed tomography showed no apparent bone loss in the palatal bones adjacent to the lesion (Figure 2).

**Figure 3:**
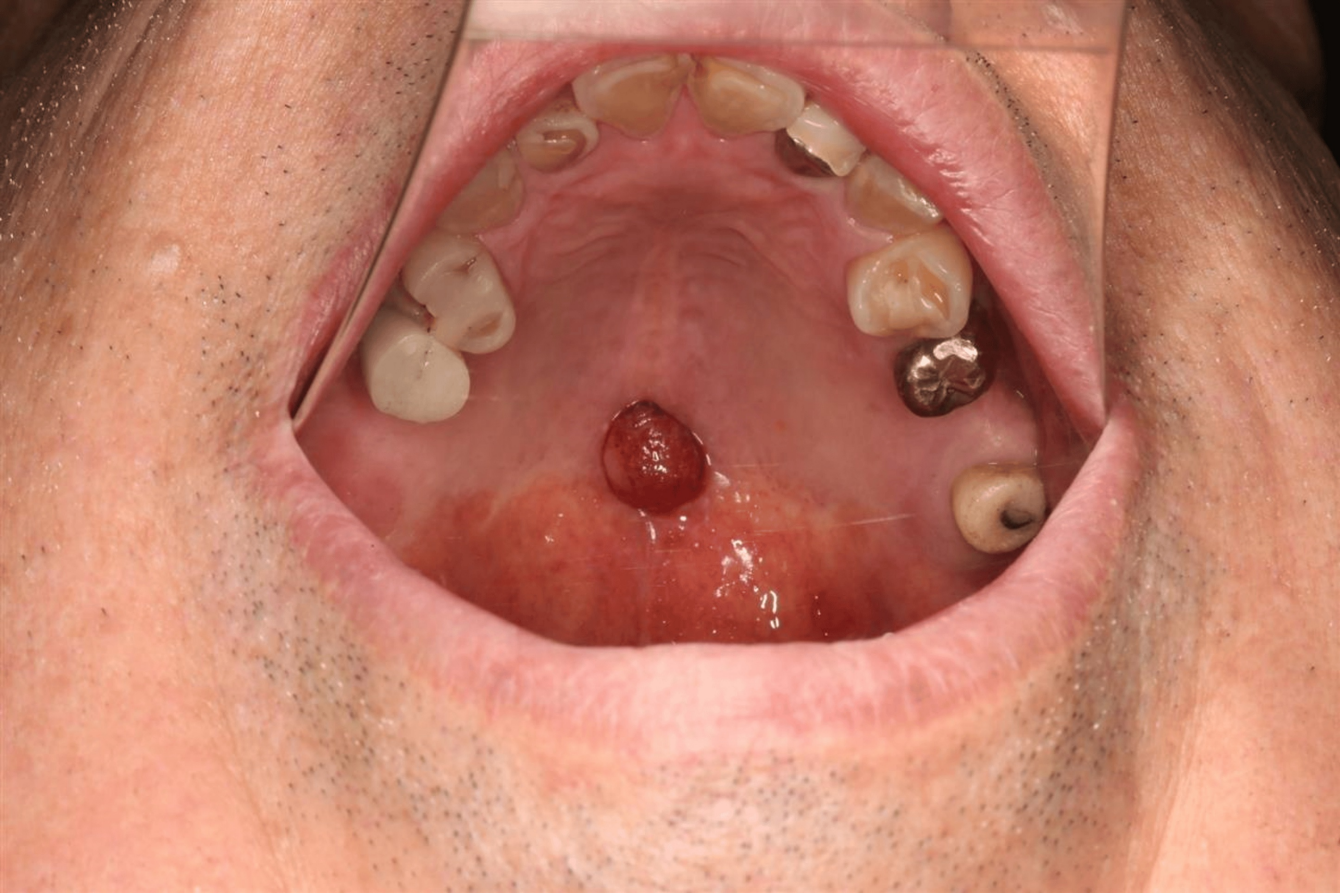
Compared to one year ago (Figure 1), the lesion has become larger

Brush cytology revealed no atypical cells and no evidence of malignancy. Therefore, the patient was diagnosed with a benign tumor of the palate, with a differential diagnosis of a minor salivary gland tumor. It would have been preferable to perform an excision biopsy while the lesion was still small to obtain a definitive diagnosis, but he did not wish to undergo surgery and preferred to be followed up. Thus, the lesion was followed up every two months for one year, but it gradually grew and showed a tendency to hemorrhage with a change in color to reddish brown (Figure 3).

**Figure 4:**
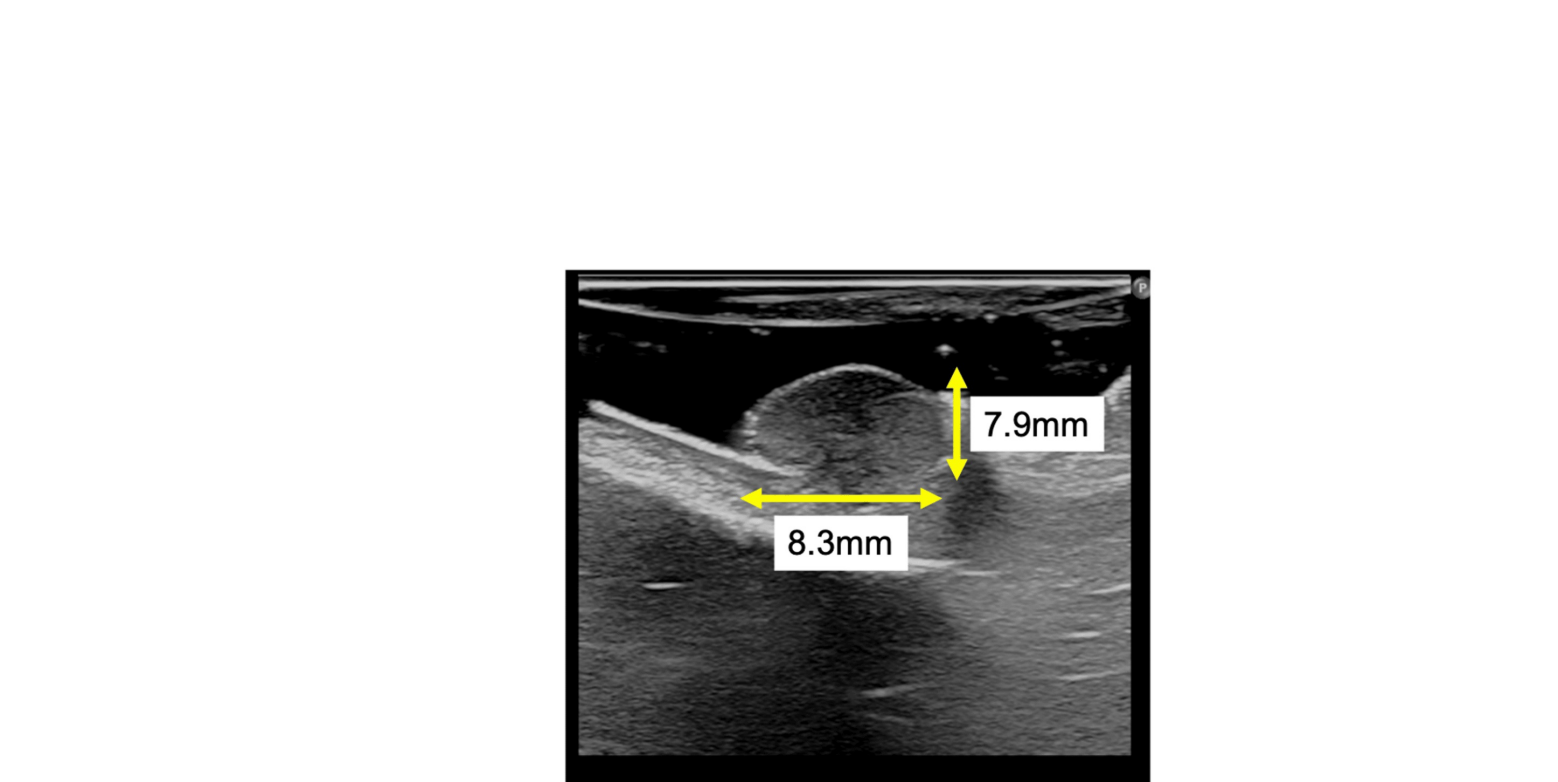
An 8.3 × 8.3 × 7.9 mm lesion shown by ultrasonography

Therefore, the patient underwent second brush cytology one year after the first and was found to have atypical cells in the lesion. Ultrasonography showed an 8.3 × 8.3 × 7.9 mm lesion with the lesion contiguous with the palatal mucosal layer and accompanied by a pale hypoechoic and pedunculated mass (Figure 4).

**Figure 5:**
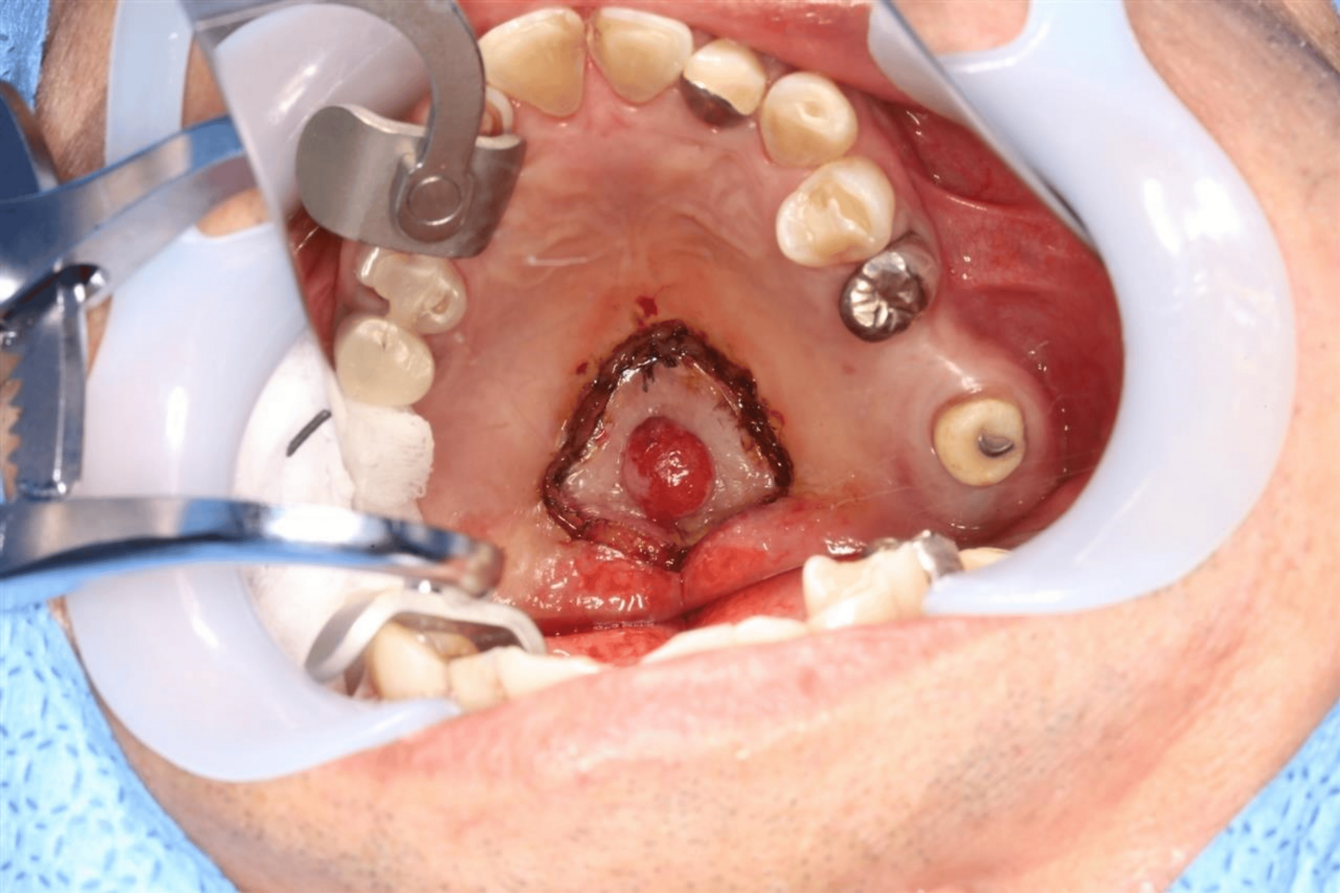
An excisional biopsy with a safety margin of 7 mm from the lesion

Additionally, ultrasonography revealed blood flow patterns similar to those of verrucous lesions. Based on these findings, the patient was diagnosed with a palatal tumor suspected to be papilloma or minor salivary gland cancer. Excisional biopsy was performed with a safety margin of 7 mm from the lesion (Figure 5).

**Figure 6:**
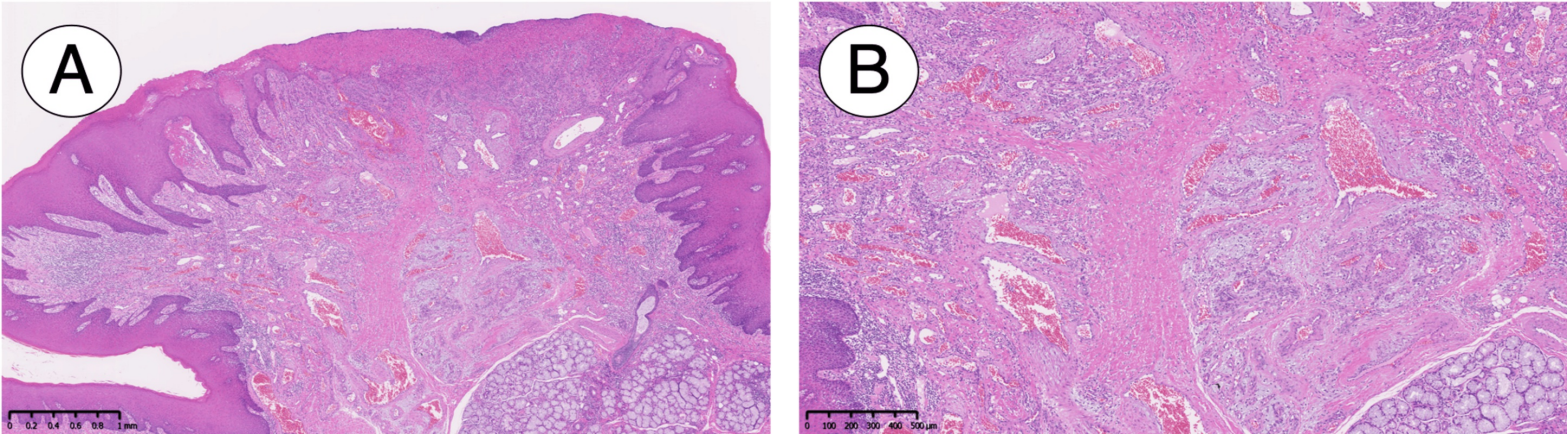
Pathological examination: (A) ×50 and (B) ×100

The lesion was easily exfoliated from the bone, and no bone destruction was observed on the exposed surface. The wound surface was then covered with a mouth guard filled with bacitracin ointment (Baramycin ointment). The pathological examination revealed a mass covered with mucosal epithelium showing a hyperkeratotic tendency, with subepithelial capillary vessels showing a lobulated growth (Figure 6).

Additionally, the specimen exhibited defects in the epithelium and its underlying connective tissue, reactive atypia characterized by nucleolus clarification, and increased nuclear size. However, no apparent malignant findings were observed, and no tumor cells were found to remain at the margins, the patient was diagnosed with pyogenic granuloma. It has now been nine months since the surgery, the patient was monitored regularly and showed no recurrence.

## Discussion

This report presents a case of a patient who exhibited pyogenic granuloma with a clinical course resembling that of a malignant tumor. Pyogenic granuloma is a nonneoplastic inflammatory reactive hyperplasia commonly observed in the soft tissues of the skin and oral mucosa [1]. Pyogenic granulomas are usually found on the lips, gingiva, and tongue. Uchiyama et al. reported that among 42 cases of pyogenic granulomas, three (7.1%) occurred on the palate, as in our case, which is considered relatively rare [3].

There are two possible etiologic factors: local factors, such as mucosal trauma (e.g., ill-fitting dentures) [6,7], and systemic factors, such as endocrine imbalance, diabetes mellitus and pregnancy [8,9]. In our case, there were no local factors, such as ill-fitting dentures, but the patient had a history of diabetes (hemoglobin A1c: 6.5) indicating that systemic factors caused immune deficiency and were likely to contribute to its occurrence. Thus far, the main pathology was thought to be the reactive proliferation of blood vessels in response to stimuli, and recent papers have suggested that the development of this disease is not related to infection [11]. Therefore, the diagnosis of “pyogenic granuloma” is not necessarily correct, and it has been proposed that diagnostic names, such as granulation tissue-type hemangiomas [12], capillary hemangiomas, and granulomas [13], should be recommended. Surgical excision is the standard treatment for pyogenic granulomas. However, recurrence is occasionally observed [8]. Therefore, resection with a margin of 2 mm from the lesion is required [14].

Of importance, when the size of the lesions gradually increases, they must be differentiated from malignant tumors [10]. In previous studies, 71.2%-74% of cases were correctly diagnosed with pyogenic granuloma based on clinical presentation [15,16]. These findings showed a high degree of agreement between clinical and histopathologic diagnosis. However, in a very small number of cases, malignant tumors were mistakenly clinically diagnosed as pyogenic granuloma. In our case, the lesion grew larger and showed a tendency to hemorrhage with a change in color to reddish brown during the one-year follow-up. Additionally, the patient suffered from a decline in liver function, which might have provoked his endocrine imbalance, and some local factors might have triggered local inflammation in the lesion, contributing to its growth. This proposal is supported because the second cytology revealed atypical cells that were not observed in the first cytology. 

Regarding palatal tumors, it has been reported that these tumors are mainly derived from minor salivary glands, namely, pleomorphic adenoma, mucoepidermoid carcinoma, adenoid cystic carcinoma, etc. Furthermore, about half of the minor salivary gland tumors are reported to be malignant [17,18]. Therefore, a suspicion of malignancy from minor salivary glands should be considered when diagnosing palatal tumors . Furthermore, even benign minor salivary gland tumors, such as pleomorphic adenoma, occasionally have incomplete capsules and are juxtaposed against surrounding tissues. Resection of a tumor without an adequate safety margin may result in incomplete tumor removal [19]. Considering the above findings and the atypical clinical course of the lesion in this report, excisional biopsy with a safety margin of 7 mm was performed. Compared with previous reports [14], the margin of resection of pyogenic granulomas in our case was relatively large. If tissue biopsy of a part of the lesion was performed before surgery and revealed pyogenic granuloma, the extent of resection could have been reduced. However, given that the patient presented with a lesion accompanied by inflammation and ulcers, reaching a definitive diagnosis based on tissue biopsy before surgery was difficult, as previously suggested [19]. Therefore, careful consideration should be given to the need for biopsy before surgery. In our case, the patient did not experience lesion recurrence after treatment. Systemic and local factors contribute to recurrence [20]. Careful follow-up and treatment of diabetes are required.

## Conclusions

Systemic and local factors contribute to the development of oral pyogenic granuloma. As shown in this case, clinicians should take the possibility of minor salivary gland tumors into consideration in treating palatal tumors including oral pyogenic granuloma.
